# The Relationship between the Ratio of Urine Osmolality to Serum Osmolality and Neurological Outcomes in Out-of-hospital Cardiac Arrest Patients

**DOI:** 10.31083/j.rcm2505157

**Published:** 2024-05-08

**Authors:** Seok Jin Ryu, Ji Ho Lee, Dong Hun Lee, Byung Kook Lee, Sung Jin Bae, Yun Hyung Choi, Won Gi Jeong

**Affiliations:** ^1^Department of Emergency Medicine, Chonnam National University Hospital, 61469 Gwangju, Republic of Korea; ^2^Department of Emergency Medicine, Chonnam National University Medical School, 61469 Gwangju, Republic of Korea; ^3^Department of Emergency Medicine, Chung-Ang University Gwangmyeong Hospital, College of Medicine, Chung-Ang University, 14353 Seoul, Republic of Korea; ^4^Department of Radiology, Chonnam National University Hwasun Hospital, Chonnam National University Medical School, 58128 Hwasun, Republic of Korea

**Keywords:** urine osmolality, serum osmolality, targeted temperature management, cardiac arrest, prognosis

## Abstract

**Background:**

Progressive ischemic brain injury after cardiac arrest can cause damage to the hypothalamic-pituitary axis, particularly the pituitary gland. This may impact serum osmolality (SOsm) and urine osmolality (UOsm) in patients who have experienced out-of-hospital cardiac arrest (OHCA). We assumed that a low ratio of UOsm to SOsm (USR) is related to poor outcomes among OHCA patients. Therefore, the present study was designed to evaluate the association between the USR within 72 h after the restoration of spontaneous circulation (ROSC) and 6-month neurological outcomes in OHCA patients.

**Methods:**

This prospective, observational study included OHCA patients with targeted temperature management at Chonnam National University Hospital in Gwangju, Korea, between January 2016 and December 2022. We collected SOsm and UOsm data at admission (T0) and 24 (T1), 48 (T2), and 72 h (T3) after ROSC. The primary outcome was a poor neurological outcome at 6 months defined by cerebral performance categories 3, 4, or 5.

**Results:**

This study included 319 patients. The mean UOsm and USRs at T0, T1, T2, and T3 of patients with poor outcomes were lower than those of patients with good outcomes. Multivariable analysis indicated that the USRs at T1 (odds ratio [OR], 0.363; 95% confidence interval [CI], 0.221–0.594), T2 (OR, 0.451; 95% CI, 0.268–0.761), and T3 (OR, 0.559; 95% CI, 0.357–0.875) were associated with a poor outcome. The areas under the receiver operating characteristic curves of USRs at T0, T1, T2, and T3 for predicting poor outcomes were 0.615 (95% CI, 0.559–0.669), 0.711 (95% CI, 0.658–0.760), 0.724 (95% CI, 0.671–0.772), and 0.751 (95% CI, 0.699–0.797), respectively.

**Conclusions:**

The USRs within 72 h of ROSC were associated with poor neurological outcomes at 6 months in OHCA patients.

## 1. Introduction

Most cardiac arrests are associated with permanent neurological injury even after the restoration of spontaneous circulation (ROSC); these sequelae can be life-threatening [1]. For patients who experience out-of-hospital cardiac arrest (OHCA) leading to coma after ROSC, neurological prognostication is necessary to inform patients’ families and to help clinicians target treatment to patients with neurological potential for recovery. Additionally, since unnecessary medical resource consumption may increase for patients with poor neurological outcomes, it is important to accurately predict neurological outcomes to efficiently use limited medical resources. Current guidelines recommend a combination of multiple diagnostic tests to predict neurological outcomes in OHCA patients, as accuracy of prognostication by any single predictor is not guaranteed [2].

Progressive hypoxic brain injury can affect a patient’s homeostasis by causing hypothalamic dysfunction, which can lead to problems with regulating electrolytes and total body water [3]. Electrolyte and fluid imbalances can affect serum osmolality (SOsm) and urine osmolality (UOsm) in OHCA patients. Several studies have used SOsm and UOsm to diagnose central diabetes insipidus (CDI) and have shown that CDI is associated with prognosis, including in terms of neurological outcomes and mortality among OHCA patients [4, 5]. These studies reported that 11–21% of OHCA patients were diagnosed as CDI, and all patients with CDI had poor neurological outcomes, while no patients with favorable prognoses developed CDI [4, 5]. Since the proportions of patients diagnosed with CDI did not exceed the proportions of patients with poor prognoses in these previous studies, the effectiveness of CDI for predicting prognosis is limited. Moreover, these previous studies required 7–8 days after ROSC to define the occurrence of CDI after ROSC [4, 5]. It is inefficient to predict a patient’s prognosis with a CDI diagnosis in that the relevant guideline recommends predicting prognosis at least 72 h after the return to normothermia [2]. Additionally, hyponatremia was more prevalent than hypernatremia after ROSC [6], complicating the diagnosis of CDI, as hypotonic polyuria is not typically suspected, making water deprivation tests or desmopressin administration tests challenging. However, previous research has indicated that high SOsm and low UOsm are useful for predicting CDI [4, 5]. Thus, the ratio between UOsm and SOsm within 3 days after ROSC may be associated with prognosis after cardiac arrest. To our knowledge, no published studies have investigated the relationship between SOsm and UOsm for its prognostic value in the context of OHCA.

We hypothesized that a low ratio of UOsm to SOsm (USR) is related with poor outcomes in OHCA patients. Therefore, the present study was designed to evaluate the association between the USR within 72 h after ROSC and 6-month neurological outcomes in OHCA patients.

## 2. Materials and Methods

### 2.1 Study Design and Population

This prospective, observational study included OHCA patients with targeted temperature management (TTM) at Chonnam National University Hospital in Gwangju, Korea, between January 2016 and December 2022. We included adult (≥18 years) OHCA patients who underwent TTM. The exclusion criteria were as follows: patients under 18 years of age, patients who discontinued TTM due to death or transfer to other hospitals, and those with missing data. This study was approved by the Chonnam National University Hospital Institutional Review Board (CNUH-2015-164). Written informed consent was obtained from all participants or their next of kin.

We maintained blood glucose levels within 80–200 mg/dL using intravenous glucose or insulin. If severe hyperglycemia (>350 mg/dL) or hypoglycemia was confirmed, additional glucose measurements were performed after injection. Unless hypoglycemia (≤70 mg/dL) was detected, we avoided glucose-containing solutions and used balanced crystalloid.

### 2.2 Data Collection

Data related to the following variables were obtained from the patients’ hospital records: sex, age, preexisting illness, body mass index, first on-scene monitored rhythm, bystander cardiopulmonary resuscitation, witnessed collapse, interval from collapse to ROSC, laboratory findings at admission (glucose level, lactate level, partial pressure of carbon dioxide [${\mathrm{PaCO}}_{2}$], and partial pressure of oxygen [${\mathrm{PaO}}_{2}$]), along with the target TTM temperature. Sequential Organ Failure Assessment (SOFA) scores were calculated within 24 h of admission [7].

SOsm and UOsm measurements were taken at admission (T0) and 24 (T1), 48 (T2), and 72 h (T3) after ROSC. The SOsm was measured using the OSMO STAION OM 6060 (Arkray Inc., Kyoto, Japan) and UOsm was measured using the Multi-Osmette 2430 (Precision Systems Inc., Natick, MA, USA). USRs were calculated by dividing UOsm by SOsm. We collected levels of glucose, sodium, potassium, and blood urea nitrogen (BUN) at T0, T1, T2, and T3. We investigated the presence of central diabetes insipidus. Central diabetes insipidus was defined when all of the following criteria were met: urine volume >50 cc/kg/day, urine osmolarity <300 mmol/L, serum osmolarity >300 mmol/L, and serum sodium >145 mEq/L.

One investigator measured the gray-to-white matter ratio (GWR) on brain computed tomography (CT) scans at admission. A board-certified neuroradiologist, blinded to the clinical outcomes, measured the hounsfield units of the corpus callosum, caudate nucleus, putamen, and posterior limb of the internal capsule. The regions were measured in circular shapes, approximately 9–12 ${\mathrm{mm}}^{2}$ in size, at level of the basal ganglia, manually. GWR = (putamen + caudate nucleus)/(corpus callosum + posterior limb of the internal capsule).

We assessed neurological outcomes 6 months after ROSC through phone interviews using the cerebral performance category (CPC) scale (CPC 1, good cerebral performance; CPC 2, moderate cerebral disability; CPC 3, severe cerebral disability; CPC 4, coma or vegetative state; or CPC 5, brain death or death) [8]. The primary outcome was a poor neurological outcome defined as CPC 3, 4, or 5.

### 2.3 Statistical Analysis

We evaluated categorical variables as frequencies and percentages, whereas continuous variables were evaluated as medians and interquartile ranges, depending on the Shapiro-Wilk test results. Categorical group data were comparatively analyzed using the χ^2^ test with a continuity correction in 2 × 2 tables. Continuous data were compared between the groups using Mann–Whitney U tests.

We conducted multivariable logistic regression analysis to identify the predictive force of SOsm and UOsm on 6-month CPC outcomes. Variables with *p*-values < 0.20 after univariable comparisons were included in the multivariable regression model. We performed a backward stepwise approach that sequentially eliminated variables with a threshold of *p* > 0.10 to build a final adjusted regression model. The Box-Tidwell test confirmed that all of the adjusted continuous variables within the model met the linearity assumption. Finally, shockable rhythm, SOFA scores, age, time to ROSC, and ${\mathrm{PaCO}}_{2}$ were identified as adjusted variables (**Supplementary Table 1**). Each of SOsm, UOsm, and USR at the respective time points was entered into the final model separately for analysis. The results of the logistic regression analysis are presented as odds ratios (ORs) and 95% confidence intervals (CIs). We assessed the predictive performance of SOsm and UOsm in the determination of 6-month neurological outcomes by analyzing the areas under the receiver operating characteristic curves. The comparison of dependent receiver operating characteristic curves was performed using the method proposed by DeLong *et al*. [9]. All analyses were carried out using predictive analytics software (PASW) Statistics for Windows, version 28.0 (SPSS, Inc., Chicago, IL, USA) and MedCalc, version 22.0 (MedCalc Software, BVBA, Ostend, Belgium). Statistical significance was set at *p* < 0.05 (two-sided).

## 3. Results

### 3.1 Patient Characteristics

**Fig. 1.**
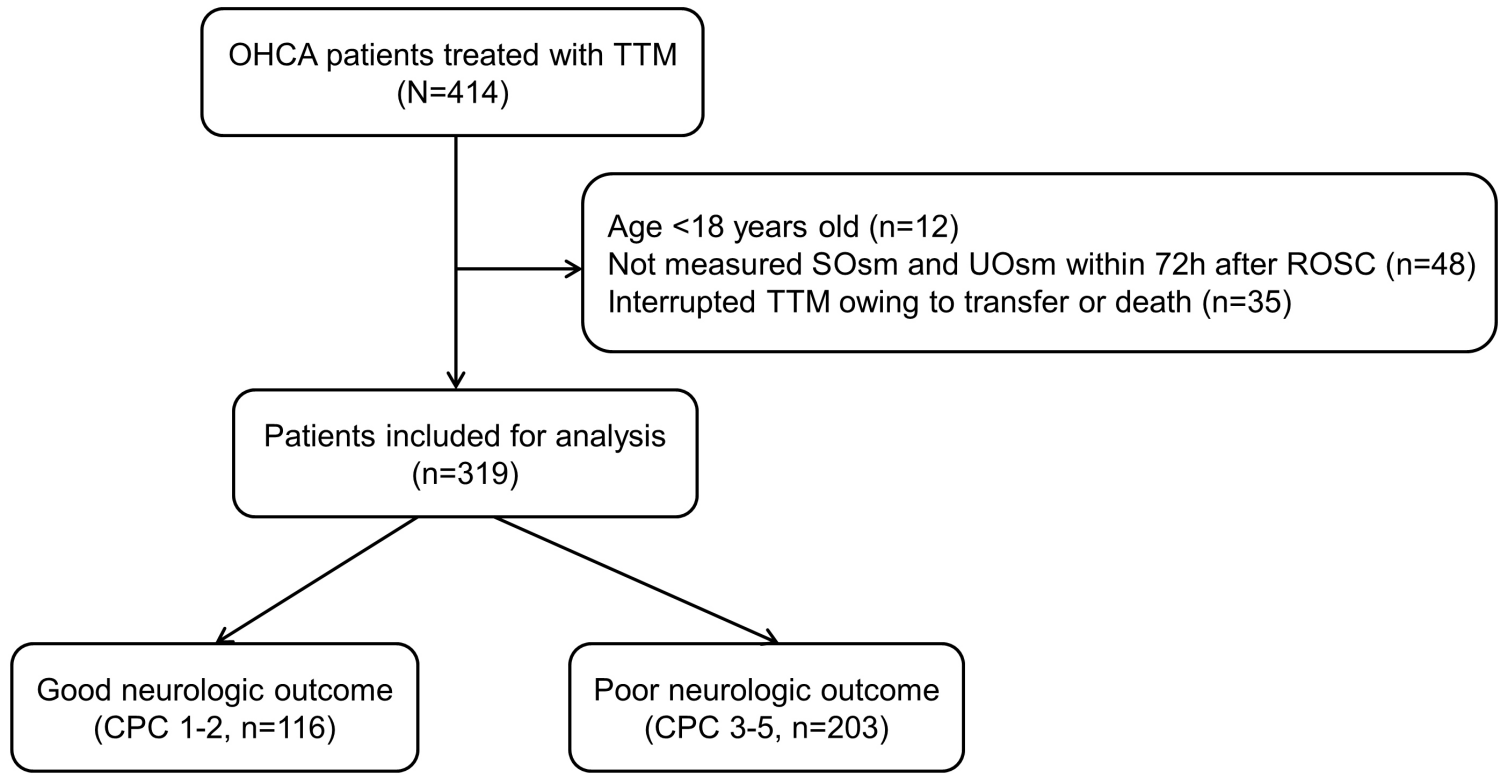
**Flow diagram of patient inclusion**. OHCA, out-of-hospital cardiac arrest; SOsm, serum osmolality; UOsm, urine osmolality; TTM, targeted temperature management; CPC, cerebral performance category; ROSC, restoration of spontaneous circulation.

**Fig. 2.**
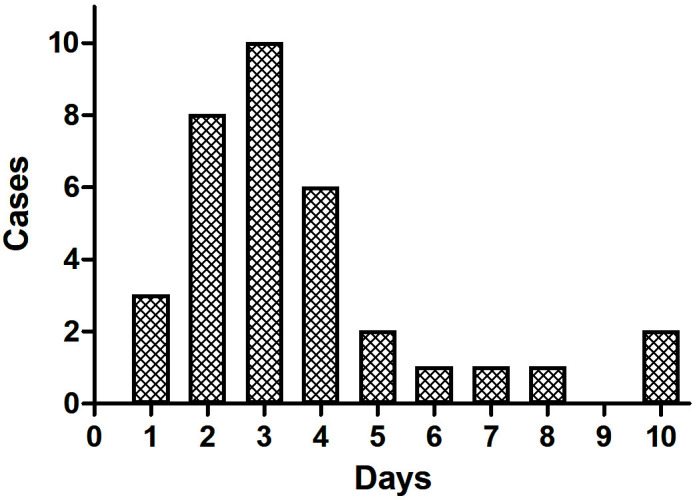
**CDI occurrence time after ROSC**. Among CDI cases, 61.7% occured within 3 days, and 38.3% occured more than 3 days after ROSC. CDI, central diabetes insipidus; ROSC, restoration of spontaneous circulation.

A total of 414 OHCA patients treated with TTM were identified during the study period. Of these, 319 patients met the inclusion criteria (Fig. 1). The median age of the OHCA patients was 61.0 years, and 241 men (75.5%) were included. In total, 210 collapses (65.8%) were witnessed by bystanders; 139 patients (43.6%) had shockable rhythms at the time of OHCA, and the mean interval from cardiac arrest to ROSC was 26.0 minutes (18.0–42.0 minutes). Patients with poor neurological outcomes had lower body mass indices, older age, and higher rates of chronic lung disease, diabetes, and hypertension than those with good neurological outcomes (Table 1). In addition, patients with poor neurological outcomes had lower rates of shockable rhythm and witnessed collapse; they also had a longer mean interval to ROSC. After ROSC, patients with poor outcomes had a lower mean Glasgow Coma Scale (GCS) score and GWR than those with favorable outcomes. The patients with poor outcomes also had a higher mean serum lactate level, mean ${\mathrm{PaCO}}_{2}$, and mean SOFA score (Table 1). CDI was present in 34 patients (10.7%), all of whom (16.7%) had poor neurological outcomes, while no patients with favorable outcomes developed CDI (Table 1). Of the CDI patients, 21 (61.7%) were diagnosed within 72 h, with the remainder diagnosed after this period (Fig. 2).

**Table 1.** **Comparisons of baseline characteristics according to neurological outcomes at 6 months**.

Variable		Total (n = 319)	Good (n = 116)	Poor (n = 203)	*p*
Demographics					
	Age, years	61.0 (49.0–71.0)	56.0 (45.0–66.0)	64.0 (53.0–74.0)	<0.001
	Male, n (%)	241 (75.5)	91 (78.4)	150 (73.9)	0.438
	Body mass index, kg/$m^{2}$	23.4 (21.0–25.7)	24.2 (22.0–26.4)	22.8 (20.4–24.8)	<0.001
Preexisting illness, n (%)					
	Coronary artery disease	56 (17.6)	19 (16.4)	37 (18.2)	0.792
	Congestive heart failure	15 (4.7)	6 (5.2)	9 (4.4)	0.980
	Hypertension	140 (43.9)	38 (32.8)	102 (50.2)	0.004
	Diabetes	86 (27.0)	16 (13.8)	70 (34.5)	<0.001
	Chronic lung disease	30 (9.4)	3 (2.6)	27 (13.3)	0.003
	Renal impairment	17 (5.3)	4 (3.4)	13 (6.4)	0.383
	Cerebrovascular accident	25 (7.8)	5 (4.3)	20 (9.9)	0.120
	Malignancy	24 (7.5)	10 (8.6)	14 (6.9)	0.733
Cardiac arrest characteristics					
	Witnessed collapse, n (%)	210 (65.8)	90 (77.6)	120 (59.1)	<0.001
	Bystander CPR, n (%)	200 (62.7)	81 (69.8)	119 (58.6)	0.061
	Shockable rhythm, n (%)	139 (43.6)	93 (80.2)	46 (22.7)	<0.001
	Interval from collapse to ROSC, min	26.0 (18.0–42.0)	19.0 (14.3–27.0)	33.0 (21.0–45.0)	<0.001
Clinical characteristics after ROSC					
	Lactate, mmol/L	7.7 (5.2–11.2)	6.4 (3.4–8.9)	9.1 (6.1–12.4)	<0.001
	Glucose, mg/dL	260 (186–326)	244 (172–303)	270 (189–333)	0.027
	${\mathrm{PaO}}_{2}$, mmHg	141.0 (89.0–233.0)	137.7 (81.1–222.5)	157.4 (92.3–248.0)	0.059
	${\mathrm{PaCO}}_{2}$, mmHg	43.0 (33.5–59.9)	38.6 (32.1–45.0)	49.6 (35.0–68.8)	<0.001
	SOFA score	11 (9–12)	10 (7–11)	11 (10–13)	<0.001
Target temperature of TTM					0.366
	33 °C, n (%)	305 (95.6%)	113 (97.4%)	192 (94.6%)	
	36 °C, n (%)	14 (4.4%)	3 (2.6%)	11 (5.4%)	
Gray-white matter ratio		1.29 (1.19–1.38), ${\mathrm{310}}^{\text{a}}$	1.35 (1.26–1.43), ${\mathrm{112}}^{\text{a}}$	1.24 (1.15–1.35), ${\mathrm{198}}^{\text{a}}$	<0.001
CDI, n (%)		34 (10.7)	0 (0.0)	34 (16.7)	<0.001

Data are presented as median (25th–75th percentile) or number (%) of patients. CPR, cardiopulmonary resuscitation; ROSC, restoration of spontaneous circulation; SOFA, Sequential Organ Failure Assessment; TTM, targeted temperature management; CDI, central diabetes insipidus; ${\mathrm{PaCO}}_{2}$, partial pressure of carbon dioxide; ${\mathrm{PaO}}_{2}$, partial pressure of oxygen. ^a^ Number included for analysis.

### 3.2 SOsm, UOsm, and USR according to Neurological Outcome at 6 Months

According to neurological outcomes at 6 months, SOsm at T0, T1, T2, and T3 among patients with poor outcomes were higher than those among patients with good outcomes. UOsm and USRs at T0, T1, T2, and T3 among patients with poor outcomes were lower than those among patients with good outcomes (Table 2). Our analysis of SOsm, UOsm, and USR values according to the target TTM temperature showed no significant differences between the target temperatures of 33 °C and 36 °C.

**Table 2.** **Comparisons of SOsm, UOsm, and USR according to neurological outcomes at 6 months and target temperature**.

Variable	Total (n = 319)	Good (n = 116)	Poor (n = 203)	*p*	33 °C (n = 305)	36 °C (n = 14)	*p*
SOsm at T0, mOsm/L	300 (293–309)	299 (292–304)	303 (294–313)	0.003	300 (294–309)	301 (290–325)	0.845
SOsm at T1, mOsm/L	296 (290–303)	293 (289–298)	298 (291–305)	<0.001	296 (290–303)	296 (289–304)	0.720
SOsm at T2, mOsm/L	295 (289–302)	292 (288–298)	297 (290–305)	<0.001	295 (289–302)	295 (290–298)	0.752
SOsm at T3, mOsm/L	297 (290–305)	293 (290–299)	300 (292–309)	<0.001	297 (291–305)	293 (287–303)	0.136
UOsm at T0, mOsm/L	389 (340–481)	428 (354–520)	381 (330–456)	0.002	392 (341–484)	369 (330–415)	0.244
UOsm at T1, mOsm/L	415 (310–593)	514 (394–671)	355 (265–527)	<0.001	415 (311–582)	358 (298–694)	0.814
UOsm at T2, mOsm/L	429 (322–606)	541 (398–710)	363 (303–519)	<0.001	431 (321–608)	424 (328–598)	0.954
UOsm at T3, mOsm/L	457 (314–655)	582 (455–733)	368 (292–540)	<0.001	452 (310–651)	471 (332–672)	0.735
USR at T0	1.29 (1.10–1.64)	1.44 (1.17–1.78)	1.24 (1.08–1.54)	<0.001	1.30 (1.11–1.64)	1.18 (1.03–1.38)	0.108
USR at T1	1.39 (1.05–1.98)	1.78 (1.35–2.30)	1.18 (0.91–1.74)	<0.001	1.40 (1.05–1.97)	1.18 (1.02–2.40)	0.822
USR at T2	1.44 (1.08–2.07)	1.85 (1.35–2.46)	1.21 (1.01–1.74)	<0.001	1.44 (1.08–2.07)	1.42 (1.12–2.02)	0.954
USR at T3	1.54 (1.05–2.23)	1.99 (1.54–2.56)	1.22 (0.99–1.86)	<0.001	1.54 (1.04–2.22)	1.60 (1.14–2.32)	0.663

Data are presented as median (25th–75th percentile). SOsm, serum osmolality; UOsm, urine osmolality; USR, ratio of UOsm to SOsm.

### 3.3 The Relationships of the SOsm, UOsm and USR for 6-Month Outcomes

After confounders were adjusted for, the UOsm values at T1 (OR, 0.997; 95% CI, 0.995–0.998), T2 (OR, 0.997; 95% CI, 0.996–0.999), and T3 (OR, 0.998; 95% CI, 0.997–1.000) were independently associated with poor outcomes (Table 3). The USRs at T1 (OR, 0.363; 95% CI, 0.221–0.594), T2 (OR, 0.451; 95% CI, 0.268–0.761), and T3 (OR, 0.559; 95% CI, 0.357–0.875) were independently associated with poor outcomes (Table 3). For SOsm, only the measurement at T3 was associated with poor outcomes (OR, 1.048; 95% CI, 1.012–1.085).

**Table 3.** **Multivariate logistic regression analysis of SOsm, UOsm, and USR for poor outcomes**.

Variable	Adjusted OR ${\mathrm{(95\% CI)}}^{\text{a}}$	*p*
SOsm at T0, mOsm/L	0.994 (0.978–1.010)	0.443
SOsm at T1, mOsm/L	1.030 (0.994–1.068)	0.108
SOsm at T2, mOsm/L	1.036 (0.998–1.076)	0.065
SOsm at T3, mOsm/L	1.048 (1.012–1.085)	0.008
UOsm at T0, mOsm/L	1.000 (0.997–1.002)	0.719
UOsm at T1, mOsm/L	0.997 (0.995–0.998)	<0.001
UOsm at T2, mOsm/L	0.997 (0.996–0.999)	0.004
UOsm at T3, mOsm/L	0.998 (0.997–1.000)	0.016
USR at T0	1.029 (0.459–2.306)	0.944
USR at T1	0.363 (0.221–0.594)	<0.001
USR at T2	0.451 (0.268–0.761)	0.003
USR at T3	0.559 (0.357–0.875)	0.011

Each of SOsm, UOsm, and USR at the respective time points was entered into the final model and analyzed. ^a^ Adjusted for age, SOFA score, interval from collapse to ROSC, shockable rhythm, and ${\mathrm{PaCO}}_{2}$ level. SOsm, serum osmolality; UOsm, urine osmolality; USR, ratio of UOsm to SOsm; SOFA, Sequential Organ Failure Assessment; ROSC, restoration of spontaneous circulation; OR, odds ratio; ${\mathrm{PaCO}}_{2}$, partial pressure of carbon dioxide.

**Fig. 3.**
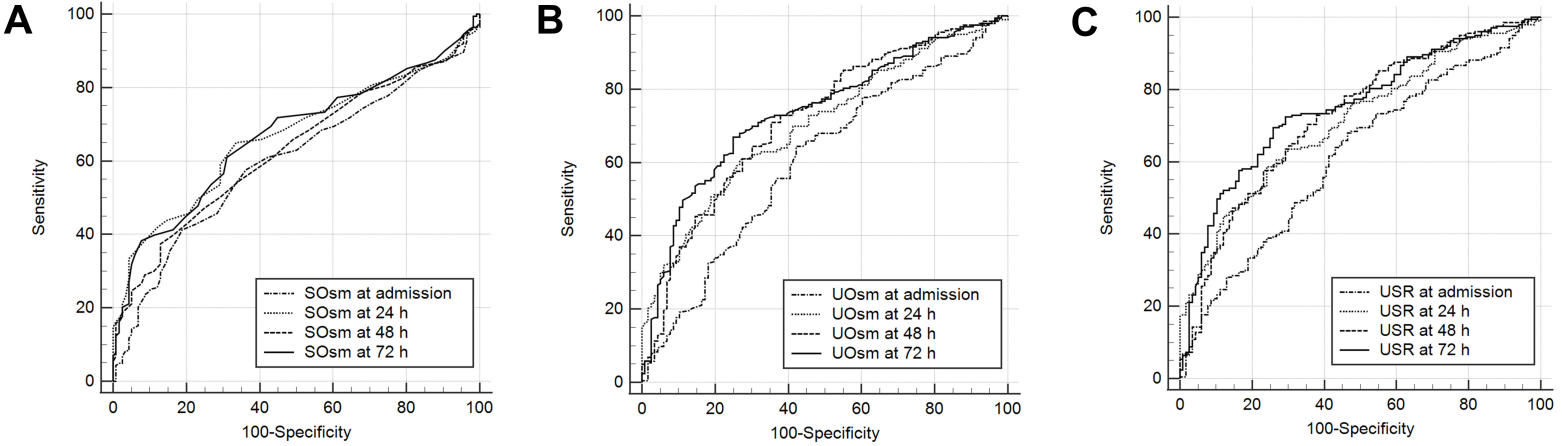
**AUCs for SOsm (A), UOsm (B), and USR (C) for predicting poor outcomes**. The AUC for USR at 72 h after ROSC was significantly different from those for SOsm and UOsm at the same time point. AUCs, areas under the receiver operating characteristic curves; SOsm, serum osmolality; UOsm, urine osmolality; USR, ratio of urine osmolality to serum osmolality; ROSC, restoration of spontaneous circulation.

The areas under the receiver operating characteristic curves (AUCs) for the SOsm, UOsm, and USRs at T0, T1, T2, and T3 for predicting poor neurological outcomes at 6 months are presented in Fig. 3. The AUCs for SOsm at T0, T1, T2, and T3 for poor outcomes were 0.601 (95% CI, 0.545–0.655), 0.666 (95% CI, 0.611–0.718), 0.628 (95% CI, 0.573–0.682), and 0.669 (95% CI, 0.614–0.720), respectively (Fig. 3A). The AUCs for UOsm at T0, T1, T2, and T3 were 0.605 (95% CI, 0.549–0.659), 0.702 (95% CI, 0.649–0.752), 0.717 (95% CI, 0.665–0.766), and 0.738 (95% CI, 0.686–0.785), respectively (Fig. 3B). The AUCs for USRs at T0, T1, T2, and T3 were 0.615 (95% CI, 0.559–0.669), 0.711 (95% CI, 0.658–0.760), 0.724 (95% CI, 0.671–0.772), and 0.751 (95% CI, 0.699–0.797), respectively (Fig. 3C). The AUC of the USR at T3 was significantly different from those of the SOsm and UOsm at T3 after ROSC.

### 3.4 Comparative Analysis according to Central Diabetes Insipidus

The patients with CDI had a higher mean SOsm, as well as lower mean UOsm and USR values than the patients without CDI (Table 4). The patients with CDI had a lower mean GWR on CT at T0 than the patients without CDI (Table 4). The patients with CDI had higher mean glucose levels at T2 and T3 than patients without CDI (**Supplementary Table 2**). The patients with CDI had higher mean sodium levels at T1, T2, and T3 than patients without CDI (**Supplementary Table 2**). Potassium and BUN levels did not differ between the patients with CDI and patients without CDI, significantly.

**Table 4.** **Comparisons of SOsm, UOSm, USR, and GWR according to presence of central diabetes insipidus**.

Variable	No CDI (n = 285)	CDI (n = 34)	*p*
SOsm at T0, mOsm/L	300 (292–308)	308 (301–323)	0.001
SOsm at T1, mOsm/L	295 (290–302)	305 (300–314)	<0.001
SOsm at T2, mOsm/L	294 (289–300)	307 (299–316)	<0.001
SOsm at T3, mOsm/L	295 (290–303)	313 (302–329)	<0.001
UOsm at T0, mOsm/L	395 (343–491)	358 (310–425)	0.017
UOsm at T1, mOsm/L	417 (320–602)	334 (177–528)	<0.009
UOsm at T2, mOsm/L	443 (329–624)	358 (194–490)	<0.003
UOsm at T3, mOsm/L	488 (325–668)	306 (189–384)	<0.001
USR at T0	1.31 (1.11–1.67)	1.16 (1.00–1.40)	<0.004
USR at T1	1.42 (1.06–2.03)	1.11 (0.58–1.74)	<0.003
USR at T2	1.52 (1.11–2.09)	1.12 (0.65–1.66)	<0.001
USR at T3	1.64 (1.09–2.27)	0.98 (0.58–1.26)	<0.001
Gray-to-white matter ratio	1.30 (1.20–1.39), ${\mathrm{197}}^{\text{a}}$	1.21 (1.14–1.35), ${\mathrm{113}}^{\text{a}}$	0.011

Data are presented as median (25th–75th percentile). SOsm, serum osmolality; UOsm, urine osmolality; USR, ratio of UOsm to SOsm; CDI, central diabetes insipidus; GWR, gray-to-white matter ratio. ^a^ Number included for analysis.

## 4. Discussion

In this prospective cohort study, the patients with poor neurological outcomes who underwent TTM after ROSC had higher SOsm, as well as lower UOsm and USRs than the patients with good neurological outcomes. USRs at T1, T2, and T3 were robustly associated with poor neurological outcomes. AUC analysis indicated that while USR at T0 was a poor predictor, USRs at T1, T2, and T3 were fair predictors of poor neurological outcomes. Among these, USR at T3 exhibited the highest performance for predicting poor outcomes.

Several studies showed CDI to be associated with mortality and poor neurological outcomes after cardiac arrest [4, 5]. The diagnosis of CDI typically requires a urine output exceeding 300 mL/h and the administration of desmopressin, a synthetic analogue of antidiuretic hormone [4, 5]. However, two previous studies [4, 5] demonstrated that the diagnosis of CDI can be confirmed through observation up to 7 days after ROSC, whereas the time required to assess the neurological status of OHCA patients is generally 72 h after the return to normothermia following ROSC. Thus, CDI diagnosis may be delayed relative to the determination of neurological prognosis. Additionally, acute kidney injury or cardiogenic shock may occur during the post-resuscitation period in OHCA patients, which may make it difficult to measure urine output for diagnosing CDI due to anuria or oliguria. However, as shown in the present study, if USR is used in the early stages rather than for confirming the presence or absence of CDI, it can provide more information for determining neurological outcomes and help medical staff make treatment decisions.

In the present study, the mean SOsm values at T1, T2, and T3 of patients with poor neurological outcomes were higher than those of patients with good neurological outcomes. Elevated osmolality has been associated with a poor prognosis in association with many conditions, such as heart failure and traumatic brain injury [10, 11, 12]. In a study investigating OHCA, the mean SOsm of patients with poor neurological outcomes was higher than that of patients with good neurological outcomes, which was consistent with our study (at 0 h, 303.5 vs. 297.3 milliosmoles (mOsm)/L; 24 h, 300.5 vs. 288.4 mOsm/L) [13]. In that study, the investigators speculated that blood–brain barrier breakdown after cardiac arrest causes sodium leakage from blood vessels into the interstitial space, ultimately aggravating cerebral edema in OHCA survivors [13]. Although that study showed a relationship between SOsm and neurological outcomes within 24 h after ROSC, in the present study, this relationship persisted until 72 h; multivariate analysis indicated that only SOsm at T3 was associated with poor neurological outcomes.

The univariate analysis revealed that SOsm, UOsm, and USR were related to poor neurological outcomes at all periods within 72 h after ROSC. However, the multivariate analysis revealed that sOsm, UOsm, and USR at admission were not associated with poor outcomes, and SOsm was associated with poor outcomes only at 72 h after ROSC. It is suggested that SOsm, UOsm, and USR may not exert a significant influence on outcomes upon admission when only ischemic injury was reflected. Their effect on outcomes becomes evident only when reperfusion injury is present in addition to ischemic injury. Furthermore, SOsm is affected by various factors, including electrolytes or blood glucose levels, and can be a target for fluid resuscitation. Thus, it would not be related to outcomes other than at 72 h after ROSC, when reperfusion injury is a prominent neurological outcome. Additionally, SOsm and UOsm at T3 were associated with poor neurological outcomes, with their predictive power enhanced when combined with USR. The AUC for USR at T3 was superior to those of SOsm and UOsm at the same time point.

Cardiac arrest induces damage to the hypothalamic–pituitary axis, and the pituitary gland is particularly vulnerable to ischemia [14], so it is expected that CDI will occur due to severe brain damage after cardiac arrest. The most typical phenomenon among CDI features is reduced UOsm. In this regard, although two previous studies did not indicate exact UOsm values corresponding to specific neurological outcomes in the post-resuscitation period [4, 5], a poor neurological prognosis can be inferred from a low UOsm. Additionally, several case reports have indicated low UOsm in patients with severe brain damage after cardiac arrest [15, 16, 17]. In our study, the median UOsm of patients with poor outcomes was lower than that of patients with good outcomes within 72 h after ROSC, and in the multivariate analysis, UOsm at T1, T2, and T3 was associated with poor neurological outcomes. Elevated serum sodium levels, another characteristic of CDI, were observed in our study, with hypernatremia (defined as a serum sodium concentration above 145 mmol/L) present at T1, T2, and T3.

Normal UOsm values may vary depending on the condition of patients, but the normal range is approximately 500–800 mOsm/L [18, 19, 20], and the mean UOsm of 380 mOsm/L immediately after ROSC in the present study was lower than normal. We thought that brain damage secondary to ischemia during cardiac arrest would lead to hypothalamic–pituitary axis compromise in most patients after ROSC. However, UOsm of patients with good outcomes gradually increased over time, whereas that of patients with poor outcomes had little change after ROSC. In other words, patients with good neurological outcomes recovered from the initial brain injury, but patients with poor neurological outcomes did not.

The present study had several limitations. First, this was a single-center observational study. Therefore, its results cannot be widely generalized. Further prospective multicenter studies are needed to complement our findings. Second, we excluded patients who discontinued TTM due to death or transfer to other hospitals. This might have led to selection bias and influenced the study outcomes. Third, given that we examined SOsm and UOsm within 72 h after ROSC in the present study, we could not evaluate the relationship between SOsm and UOsm beyond 72 h and neurological prognoses. Fourth, the effects of fluids and drugs on SOsm and UOsm during the post-resuscitation period were not considered. Although efforts were made to keep glucose levels within an optimal range, the administration of glucose-containing fluids or insulin could alter SOsm and UOsm. Additionally, desmopressin, an antidiuretic medication, may be administered based on clinical suspicion of CDI, further influencing osmolality measurements. Future research should investigate the relationships between these medications and osmolality.

## 5. Conclusions

USRs within 72 h of ROSC were associated with poor neurological outcomes at 6 months after OHCA. USR may serve as a valuable indicator, in conjunction with other prognostic factors, to identify patients with severe conditions and to guide the administration of more intensive treatments post-ROSC.

## Data Availability

All data generated or analyzed during this study are included in this article and its supplementary material files. Further enquiries can be directed to the corresponding author.

## Supplementary Material

Supplementary material associated with this article can be found, in the online version, at https://doi.org/10.31083/j.rcm2505157.
