# Supplementary material for: The Relationship between the Ratio of Urine Osmolality to Serum Osmolality and Neurological Outcomes in Out-of-hospital Cardiac Arrest Patients
Source: Rev Cardiovasc Med. 2024 May 8;25(5):157. doi: 10.31083/j.rcm2505157 (PMC11267209; doi:10.31083/j.rcm2505157)
Supplement: Supplementary file 1 [file 2153-8174-25-5-157-s1.docx]

Supplementary Table 1. Multivariate logistic regression analysis for poor neurologic outcomes at 6-month

| Variable | Adjusted OR (95% CI) | P |
| --- | --- | --- |
| Age, years | 1.041 (1.019–1.064) | <0.001 |
| Body mass index, kg/m^2^ | 0.943 (0.866–1.026) | 0.170 |
| Hypertension | 1.303 (0.596–2.848) | 0.507 |
| Diabetes | 2.157 (0.946–4.918) | 0.068 |
| Chronic lung disease | 1.937 (0.454–8.272) | 0.372 |
| Cerebrovascular accident | 2.727 (0.755–9.853) | 0.126 |
| Witnessed collapse, n (%) | 0.704 (0.338–1.464) | 0.347 |
| Bystander CPR, n (%) | 0.552 (0.267–1.140) | 0.108 |
| Shockable rhythm, n (%) | 0.102 (0.053–0.196) | <0.001 |
| Time to ROSC, min | 1.055 (1.032–1.079) | <0.001 |
| Lactate, mmol/L | 1.087 (0.999–1.182) | 0.054 |
| Glucose, mg/dL | 0.997 (0.994–1.000) | 0.063 |
| PaCO_2_, mmHg | 1.026 (1.005–1.047) | 0.014 |
| SOFA score | 1.210 (1.067–1.372) | 0.003 |

OR, odds ratio; CI, confidence interval; ROSC, restoration of spontaneous circulation; PaCO2, partial pressure of carbon dioxide; SOFA, Sequential Organ Failure Assessment

Supplementary Table 2. Comparisons of glucose, sodium, potassium, and BUN levels according to neurological outcomes at 6 months and CDI.

| Variable | Total (N = 319) | Good (n = 116) | Poor (n = 203) | P | No CDI (n = 285) | CDI (n = 34) | P |
| --- | --- | --- | --- | --- | --- | --- | --- |
| Glucose at T0, mg/dL | 260 (186–326) | 244 (172–303) | 270 (189–333) | 0.027 | 254 (183–323) | 274 (212–386) | 0.107 |
| Glucose at T1, mg/dL | 133 (110–169) | 125 (106–155) | 137 (115–177) | 0.025 | 133 (110–168) | 134 (107–183) | 0.926 |
| Glucose at T2, mg/dL | 132 (111–165) | 127 (108–155) | 135 (112–172) | 0.021 | 130 (109–165) | 152 (125–190) | 0.010 |
| Glucose at T3, mg/dL | 143 (117–186) | 130 (112–151) | 154 (121–202) | <0.001 | 142 (116–183) | 182 (131–233) | 0.008 |
| Sodium at T0, mmol/L | 141 (138–143) | 141 (138–143) | 140 (138–143) | 0.400 | 140 (138–143) | 141 (139–145) | 0.112 |
| Sodium at T1, mmol/L | 143 (140–145) | 143 (141–145) | 143 (140–146) | 0.305 | 143 (140–145) | 147 (145–151) | <0.001 |
| Sodium at T2, mmol/L | 143 (140–146) | 143 (140–145) | 143 (140–146) | 0.449 | 142 (140–145) | 149 (146–151) | <0.001 |
| Sodium at T3, mmol/L | 144 (141–147) | 144 (142–146) | 144 (140–147) | 0.913 | 144 (141–146) | 150 (146–154) | <0.001 |
| Potassium at T0, mmol/L | 3.9 (3.5–4.4) | 3.8 (3.5–4.3) | 4.0 (3.6–4.5) | 0.115 | 3.9 (3.5–4.4) | 3.8 (3.5–4.4) | 0.705 |
| Potassium at T1, mmol/L | 3.9 (3.5–4.3) | 3.9 (3.5–4.2) | 3.9 (3.6–4.4) | 0.086 | 3.9 (3.6–4.3) | 3.9 (3.5–4.5) | 0.889 |
| Potassium at T2, mmol/L | 4.1 (3.7–4.4) | 4.0 (3.6–4.3) | 4.2 (3.8–4.7) | <0.001 | 4.1 (3.7–4.4) | 4.0 (3.7–4.8) | 0.465 |
| Potassium at T3, mmol/L | 3.8 (3.4–4.1) | 3.7 (3.4–3.9) | 3.8 (3.5–4.3) | <0.001 | 3.8 (3.4–4.1) | 3.8 (3.6–4.2) | 0.294 |
| BUN at T0, mg/dL | 18.9 (15.1–24.8) | 18.3 (15.3–21.9) | 19.3 (15.0–26.8) | 0.080 | 19.1 (15.2–25.1) | 17.3 (13.8–23.0) | 0.157 |
| BUN at T1, mg/dL | 19.9 (14.2–29.9) | 16.3 (11.4–22.4) | 24.2 (16.4–33.3) | <0.001 | 19.7 (14.2–29.7) | 24.7 (14.8–31.8) | 0.311 |
| BUN at T2, mg/dL | 20.4 (13.3–29.7) | 15.6 (11.4–22.5) | 24.2 (15.8–33.2) | <0.001 | 20.0 (13.1–29.7) | 24.4 (14.1–32.2) | 0.449 |
| BUN at T3, mg/dL | 20.8 (13.2–29.9) | 15.4 (10.8–23.3) | 23.0 (15.4–36.0) | <0.001 | 20.7 (13.6–29.8) | 21.9 (11.2–30.8) | 0.799 |

Data are presented as median (25th–75th percentile).

CDI, central diabetes insipidus; BUN, blood urea nitrogen
